# Prevalence of High and Moderate Risk of Liver Fibrosis Among Patients With Diabetes at a Noncommunicable Diseases (NCD) Clinic in a Primary Healthcare Center in Northern India

**DOI:** 10.7759/cureus.49286

**Published:** 2023-11-23

**Authors:** Anubhav Mondal, Aninda Debnath, Ghurumourthy Dhandapani, Abhishek Sharma, Shveta Lukhmana, Geeta Yadav

**Affiliations:** 1 Community Medicine, Vardhman Mahavir Medical College and Safdarjung Hospital, New Delhi, IND

**Keywords:** fibrosis-4 (fib-4) score, nonalcoholic fatty liver disease (nafld), diabetes type 2, non communicable disease (ncd), liver fibrosis

## Abstract

Background

Diabetes is a known entity that contributes to increased incidence and progress of liver fibrosis. Despite the integration of nonalcoholic fatty liver disease (NAFLD) into the NP-NCD program (National Programme for Prevention and Control of Cancer, Diabetes, Cardiovascular Diseases, and Stroke [NPCDCS]), screening individuals in primary healthcare settings for liver fibrosis remains uncommon. The objective of this study was to determine the prevalence of the risk of liver fibrosis in individuals with diabetes.

Methodology

The secondary data analysis was conducted among patients with diabetes attending the noncommunicable diseases (NCD) clinic at the Primary Health Center (PHC) Najafgarh, Delhi, from January 2023 to June 2023. We used the Fibrosis-4 (FIB-4) score to assess the risk of liver fibrosis. The data analysis was carried out using Stata 17.0 software (StataCorp, College Station, TX).

Results

Out of 394 individuals screened, 158 (39.5%) were male and 236 (60.5%) were female. Among the study participants, 64.9% (95% confidence interval [CI] 60.0%-69.7%) were of low risk, 30.5% (95% CI 25.9%-35.3%) were of intermediate risk, and 4.6% (95% CI 2.7%-7.1%) were of high risk of developing liver fibrosis based on FIB-4 scoring. The increased risk was associated with increased age, duration of diabetes, and dyslipidemia.

Conclusions

The prevalence of high risk of liver fibrosis among patients with diabetes was 4.6% (95% CI 2.7%-7.1%), whereas an intermediate risk of developing liver fibrosis was observed in 30.5%. The study advocates integrating these screening tools into primary healthcare settings, alleviating the strain on larger healthcare facilities. It also underscores the importance of early detection and management of liver fibrosis in patients with diabetes.

## Introduction

South Asia, which is home to over a quarter of the world's population, is currently going through an epidemiological transition, with an increased frequency of noncommunicable diseases (NCDs). India, the region's largest country, is also a major contributor to the NCD burden. Several research undertaken over the last two decades have demonstrated that diabetes, hypertension, and dyslipidemia have a high total burden in India. Diabetes mellitus has a prevalence of 11.4% in India, which is higher than previously reported [1].

Nonalcoholic fatty liver disease (NAFLD) is the accumulation of increased fat (>5%) in the liver that is not caused by alcohol [2]. In India, NAFLD has emerged as a main cause of cirrhosis, hepatocellular carcinoma (HCC), and liver transplant [3]. Given India's massive population, the burden of NAFLD is likely to be substantial, placing strain on the country's limited healthcare resources. It can lead to an array of complications, including liver fibrosis, chronic liver disease, and liver cancer.

NAFLD and type 2 diabetes (T2D) are closely associated phenomena [2,4,5]. NAFLD may be considered as a hepatic manifestation of metabolic syndrome [6,7]. T2D mellitus (T2DM) and NAFLD share insulin resistance as a pathophysiological mechanism, and one of these disorders influences the development of the other. The presence of NAFLD with T2D raises the chance of developing complications such as nonalcoholic steatohepatitis (NASH) and fibrosis substantially more than the presence of NAFLD without chronic hyperglycemia [8].

The damage to the liver due to fibrosis is irreversible, and hence, it becomes impertinent to halt the progression of the disease as early as possible [9]. Thus, screening for liver fibrosis at an early stage becomes necessary to intervene early and stop the complications. Currently, the only reliable way to confirm liver fibrosis is liver biopsy, an expensive and invasive test that is not acceptable for screening purposes. Fibro scans are also widely used to look for liver fibrosis, as it is noninvasive and widely accepted by patients. However, due to the high costs involved, the requirement of skilled training for usage, and limited availability, it cannot be used at a primary care level in a developing country like India. Thus, a minimally invasive method is needed to screen the population for liver fibrosis [10].

In India, studies are very limited, which have seen the prevalence of liver fibrosis among patients with diabetes. The objective of this study was to estimate the prevalence of liver fibrosis among patients with diabetes attending a primary healthcare center and identify factors associated with the risk of liver fibrosis in this population.

## Materials and methods

Study setting

The research took place at the NCD clinic within the Primary Health Center (PHC) in Najafgarh. This PHC is part of the Rural Health Training Center (RHTC) in Najafgarh, situated in the Southwest Delhi district of the National Capital Territory of Delhi, India. PHC Najafgarh serves a population of 80,000. In the NCD clinic, there is a digital NCD registry that encompasses all individuals seeking treatment for diabetes and hypertension. This registry serves as a comprehensive record of patients with these NCDs.

Study population

This was a secondary data analysis. Patients with diabetes were identified, and their data were retrieved from the NCD register, at PHC Najafgarh, New Delhi. Patients with T2D who attended the NCD clinic during the study period (July 2022 to June 2023) were included in our study. We excluded individuals who had previously been diagnosed with liver-related conditions, such as viral hepatitis, autoimmune hepatitis, hemochromatosis, primary biliary cholangitis, Wilson’s disease, sclerosing cholangitis, biliary obstruction, and alpha-1 antitrypsin deficiency, as well as those with viral infections (Hepatitis B Virus [HBV] and/or Hepatitis C Virus [HCV]) or a history of alcohol use. In addition, we excluded participants with the following conditions: cancer, severe heart failure (New York Heart Association Classes III-IV), pregnancy, end-stage kidney disease, and those taking medications known to promote fatty liver disease (e.g., estrogens, amiodarone, steroids, and tamoxifen). Patients who did not have pertinent data in the patient registry were excluded from the study.

Study tool

In our study, we collected a comprehensive data set encompassing a range of clinical and laboratory data. All the laboratory tests were conducted in the laboratory of RHTC, Najafgarh. This data set included demographic variables such as age and sex, alongside pertinent health-related information such as body mass index (BMI) and the presence of comorbidities like arterial hypertension. Additionally, we recorded several key laboratory parameters, including glycated hemoglobin (HbA1c), serum alanine aminotransferase (ALT), aspartate aminotransferase (AST), total cholesterol, high-density lipoprotein (HDL) cholesterol, low-density lipoprotein (LDL) cholesterol, triglycerides, platelet (PLT) count, and creatinine levels.

To evaluate the degree of liver fibrosis in our cohort, we used the Fibrosis-4 (FIB-4) scoring system. FIB-4 is a screening tool to evaluate the risk of liver fibrosis. This system factors in the patient's age, PLT count, as well as AST and ALT levels. The FIB-4 score was calculated according to the following equation:

 FIB-4 = (age × AST)/(platelet count × √ALT)

FIB-4 scores were classified into three categories: FIB-4 < 1.30, indicating a low risk of advanced fibrosis; FIB-4 > 2.67, signifying a high risk of advanced fibrosis; and FIB-4 values between 1.30 and 2.67, representing an intermediate risk of advanced fibrosis. Notably, FIB-4 scores also exhibited strong predictive values, with FIB-4 ≥ 2.67 having an 80% positive predictive value and FIB-4 ≤ 1.30 boasting a 90% negative predictive value. In our fibrosis assessment, we categorized individuals with high and intermediate FIB-4 scores as positive for fibrosis, while those with low-risk FIB-4 scores were considered negative for advanced fibrosis [11].

Outcome

The primary objective of our study was to determine the prevalence of the risk of liver fibrosis in individuals with diabetes. As a secondary aim, we sought to investigate potential associations between sociodemographic factors and biochemical markers among patients with diabetes and their susceptibility to liver fibrosis. Patients identified with a moderate or high risk of liver fibrosis were referred to a higher-level healthcare facility for further evaluation and management.

Statistical analysis

In this study, continuous variables were described either as the median and interquartile range (IQR) or as the mean and standard deviation (SD). For discrete variables, frequency and percentage were used. To assess the relationships between categorical variables, a chi-square test was used. The prevalence of the risk of fibrosis was reported as a proportion along with a 95% confidence interval (CI). For the secondary objective of the study, both univariate and multivariable logistic regression analyses were conducted. In the univariate analysis, independent variables with a significance level of *P* ≤ 0.20 were included in the multivariate analysis. A *P*-value of <0.05 is considered statistically significant.

Ethical clearance

The study was conducted within the ethical boundaries of the Declaration of Helsinki. Permission for using the NCD data set was sought from pertinent authorities. 

## Results

**Figure 1:**
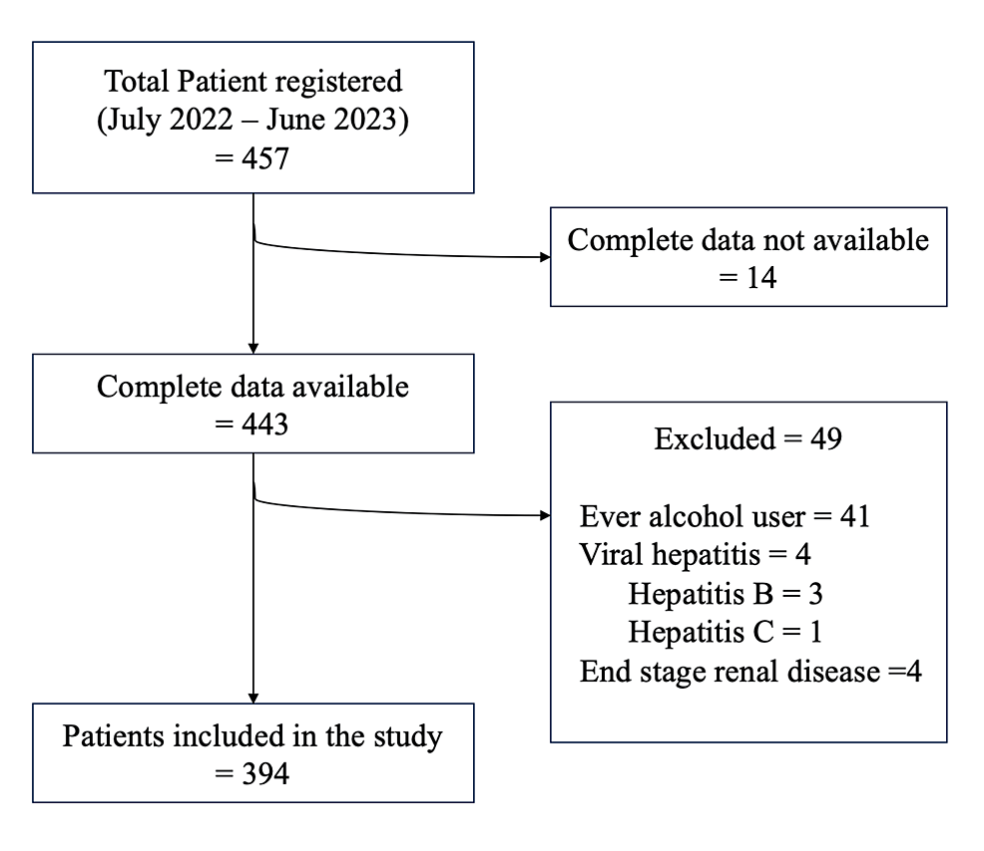
Study participant recruitment flowchart.

During the study period, a total of 457 patients with diabetes were registered in the NCD clinic. Complete data were not available for 14 (3.1%) patients. Among the remaining 443 patients, 49 (11.1%) were excluded, with 41 (83.6%) being ever-users of alcohol, 4 (8.2%) suffering from viral hepatitis, and 4 (8.2%) having end-stage renal disease (ESRD) (Figure 1). The remaining 394 eligible patients with diabetes were screened for the risk of liver fibrosis. Among the participants, the majority were female (239, 60.4%). The mean age of the study participants was 53.4 ± 10.1 years. The majority of the study participants (246, 62.4%) were obese. While almost half of the subjects (200, 49.5%) were known cases of hypertension, around 13.2% (*n *= 52) reported consuming tobacco products (Table 1).

**Table 1:** Sociodemographic and biochemical parameter details of the participants (N = 394). ^*^These variables are expressed as mean ± standard deviation (SD). ^$^These variables are expressed as proportions. BMI, body mass index; HbA1c, glycated hemoglobin; LDL, low-density lipoprotein; HDL, high-density lipoprotein; SGOT, serum glutamic-oxaloacetic transaminase; SGPT, serum glutamic pyruvic transaminase

	Male (*n* = 158)	Female (*n* = 236)	Total (*N* = 394)
Age*	53.3 (11.3)	53.4 (9.2)	53.4 (10.1)
Age category^$^			
Less than 30 years	2 (100%)	0 (0%)	2
30-45 years	42 (42%)	58 (58%)	100
45-60 years	68 (35.1%)	126 (64.9%)	194
More than 60 years	46 (46.9%)	52 (52.1%)	98
BMI^$^			
Underweight	6 (60%)	4 (40%)	10
Normal	32 (51.6%)	30 (48.4%)	62
Overweight	44 (57.9%)	32 (42.1%)	76
Obese	76 (30.9%)	170 (69.1%)	246
Hypertension^$^			
Yes	84 (42.4%)	114 (57.6%)	195
No	74 (33.8%)	122 (67.2%)	200
Tobacco^$^			
Yes	46 (88.5%)	6 (11.5%)	52
No	112 (32.8%)	230 (67.2%)	342
HbA1c*	6.9 ± 1.9	6.6 ± 1.7	6.8 ± 1.8
LDL*	102.9 ± 42.9	117.1 ± 40.1	111.4 ± 41.8
HDL*	43.5 ± 7.4	46.4 ± 7.1	45.2 ± 7.3
Total cholesterol*	177.7 ± 44.5	195.6 ± 48.4	188.4 ± 47.7
Triglyceride*	167.6 ± 80.1	166.1 ± 94.1	166.7 ± 88.6
SGOT*	35.4 ± 29.1	28.2 ± 18.4	31.1 ± 23.6
SGPT*	45.6 ± 36.8	36.3 ± 20.9	40.1 ± 28.7
Platelet*	225.1 ± 68	219.7 ± 62.9	221.5 ± 65.0
Creatinine^*^	1.03 ± 0.3	1.9 ± 2.6	1.6 ± 2.6

**Figure 2:**
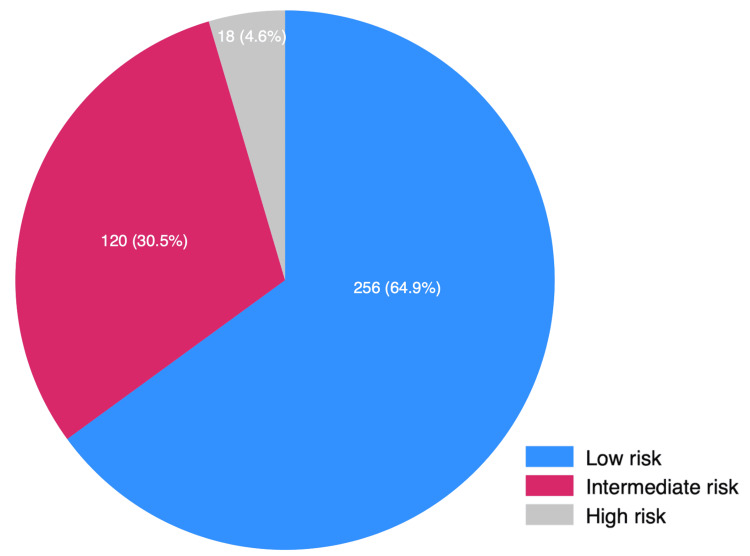
Prevalence of risk of liver fibrosis among patients with diabetes.

Among the study participants, 64.9% (*n *= 256, 95% CI 60.0%-69.7%) were of low risk, 30.5% (*n *= 120, 95% CI 25.9%-35.3%) were of intermediate risk, and 4.6% (*n *= 18, 95% CI 2.7%-7.1%) were of high risk of developing liver fibrosis according to FIB-4 scoring (Figure 2).

As demonstrated in Table 2, association was present between age and risk of liver fibrosis. The risk of liver fibrosis increases with increasing age. Duration of diabetes was also associated (*P* = 0.01, statistically significant) with the risk of liver fibrosis. Those who had a duration of more than five years had a higher risk of liver fibrosis. Dyslipidemia was also associated with the risk of fibrosis. Participants with raised LDL (*P *= 0.01, statistically significant), HDL (*P *= 0.01, statistically significant), and total cholesterol (*P* = 0.03, statistically significant) had a higher risk of developing risk of liver fibrosis.

**Table 2:** Association of sociodemographic profile and biochemical profile with the risk of liver fibrosis among patients with diabetes. ^*^Statistically significant (*P* <0.05). BMI, body mass index; HbA1c, glycated hemoglobin; LDL, low-density lipoprotein; HDL, high-density lipoprotein; SGOT, serum glutamic-oxaloacetic transaminase; SGPT, serum glutamic pyruvic transaminase

		No risk of fibrosis	Intermediate/High risk of fibrosis	Total	*P*-value
Gender	Male	98 (62%)	60 (38%)	158 (100%)	0.31
	Female	158 (66.9%)	78 (33.1%)	236 (100%)	
Age category	Less than 30 years	2 (100%)	0 (0%)	2 (100%)	0.01*
	30-45 years	84 (84%)	16 (16%)	100 (100%)	
	45-60 years	126 (64.9%)	68 (35.1%)	194 (100%)	
	More than 60 years	44 (44.9%)	54 (55.1%)	98 (100%)	
BMI	Underweight	4 (40%)	6 (60%)	10 (100%)	0.58
	Normal	34 (54.8%)	28 (45.2%)	62 (100%)	
	Overweight	48 (63.2%)	28 (36.8%)	76 (100%)	
	Obese	170 (69.1%)	76 (30.9%)	246 (100%)	
Hypertension	Yes	128 (64.6%)	70 (35.4%)	198 (100%)	0.89
	No	128 (65.3%)	68 (34.7%)	196 (100%)	
Tobacco	Yes	34 (65.4%)	18 (34.6%)	52 (100%)	0.94
	No	218 (64.9%)	118 (35.1%)	336 (100%)	
HbA1c	Normal	186 (64.6%)	102 (35.4%)	288 (100%)	0.78
	Abnormal	70 (66%)	36 (44%)	106 (100%)	
Duration of diabetes	Less than five years	200 (69.4%)	88 (30.6%)	288 (100%)	0.01*
	More than five years	55 (52.4%)	50 (47.6%)	105 (100%)	
	Oral hypoglycemic drugs	247 (65.5%)	130 (34.5%)	377 (100%)	0.28
	Oral hypoglycemic drugs and insulin	9 (52.9%)	8 (47.1%)	17 (100%)	
LDL	Normal	241 (67.7%)	115 (32.3%)	356 (100%)	0.01*
	Abnormal	15 (39.4%)	23 (60.6%)	38 (100%)	
HDL	Normal	212 (67.9%)	100 (32.1%)	312 (100%)	0.01*
	Abnormal	44 (53.7%)	38 (46.3%)	82 (100%)	
Total cholesterol	Normal	176 (68.8%)	80 (31.2%)	256 (100%)	0.03*
	Abnormal	80 (58%)	58 (42%)	138 (100%)	
Triglyceride	Normal	204 (65.8%)	106 (34.2%)	310 (100%)	0.50
	Abnormal	52 (61.9%)	32 (38.1%)	84 (100%)	
Creatinine	Normal	242 (65.1%)	130 (34.9%)	372 (100%)	0.89
	Abnormal	14 (63.6%)	8 (36.4%)	22 (100%)	

Further, on performing multivariable logistic regression, the association of high or moderate risk of fibrosis was found with age, duration of diabetes, LDL, and HDL. The odds of having a higher risk of developing fibrosis was 3.2 times in the age group of 45 to 60 years compared to the participants who were less than 45 years old. The odds were even higher for those who were aged more than 60 years. The participants who had diabetes for a longer during had 1.9 (95% CI 1.2-3.2) times more risk of developing liver fibrosis compared to those who had diabetes for less than five years. Those with dyslipidemia had a higher risk. Those with deranged LDL had 2.3 (95% CI 1.1-4.8) times higher risk, and those with deranged HDL had 2.3 (95% CI 1.3-4.1) times higher risk of developing fibrosis (Table 3).

**Table 3:** Univariate and multivariable logistic regression analyses to find the association between sociodemographic, disease, and biochemical profiles with the risk of liver fibrosis among patients with diabetes. ^*^Statistically significant (*P *< 0.05). ^#^Group that was referenced for odds ratio calculation. CI, confidence interval; LDL, low-density lipoprotein; HDL, high-density lipoprotein

	Unadjusted odds ratio (95% CI)	*P*-value	Adjusted odds ratio (95% CI)	*P*-value
Age category				
Less than 45 years	Reference^#^	0.01*	Reference^#^	0.01*
45-60 years	2.9 (1.5-5.3)		3.2 (1.7-6.6)	
More than 60 years	6.5 (3.4-12.8)		7.3 (3.4-9.8)	
Duration of diabetes mellitus				
Less than five years	Reference^#^	0.01*	Reference^#^	0.01*
More than five years	2.1 (1.3-3.2)		1.9 (1.2-3.2)	
LDL				
Normal	Reference^#^	0.01*	Reference^#^	0.01*
Abnormal	3.2 (1.6-6.4)		2.3 (1.1-4.8)	
HDL				
Normal	Reference^#^	0.01*	Reference^#^	0.01*
Abnormal	1.8 (1.1-3.0)		2.3 (1.3-4.07)	
Total cholesterol				
Normal	Reference^#^	0.03*	Reference^#^	0.06
Abnormal	1.6 (1.0-2.4)		1.1 (0.7-1.8)	

## Discussion

In this study, the prevalence of high risk of liver fibrosis among patients with diabetes was 4.6% (*n *= 18, 95% CI 2.7%-7.1%), whereas intermediate risk of developing liver fibrosis was observed in 30.5% (*n *= 120, 95% CI 25.9%-35.3%) of patients with diabetes. The increased risk was associated with increased age, duration of diabetes, and dyslipidemia.

In our investigation, we utilized the FIB-4 risk scoring system as a tool to estimate the likelihood of liver fibrosis among individuals with known diabetes. Notably, this study stands as the pioneering effort in India to gauge the risk of liver fibrosis in patients with diabetes through a questionnaire-based methodology that can be used in low-resource settings. When combined, the cumulative prevalence of high and moderate risk for developing liver fibrosis in this study was 35.1% (95% CI 30.2-39.8). Comparative studies in India have addressed this concern as well. For instance, a study conducted by Sinha and Bankura, using transient elastography, found the risk of moderate-to-severe liver fibrosis to be 32% [12]. Another study within the population with diabetes in India indicated a prevalence of clinically relevant liver fibrosis at 28% [13]. On the global front, various studies have used the FIB-4 score to assess liver fibrosis risk among individuals with diabetes. In a study conducted in Japan, a high or intermediate risk of liver fibrosis was noted in 59.6% of cases [14]. Similarly, research by Chung et al. in South Korea revealed that 43% of individuals with diabetes had an intermediate to high risk of liver fibrosis [15]. Further afield, a study in the United Kingdom by Claudia et al. disclosed that 75.2% of subjects fell within the low-risk FIB-4 score range, 22.3% within the intermediate range, and 2.5% were classified in the high-risk category [16]. In the United States, among 447 individuals with diabetes, 4% reported high FIB-4 scores [17]. These variations in prevalence can be attributed to differences in population demographics and the specific settings of each study.

Our study shows a significant increase in the risk of fibrosis with increasing age. This is in line with previously published studies [18]. This observed age-dependent susceptibility to fibrosis may be attributed to the liver's diminishing capacity to regenerate and recuperate from inflammatory insults as individuals grow older, potentially heightening the risk of fibrosis development [19]. Furthermore, our research unveiled a significant association between the duration of diabetes and the likelihood of liver fibrosis. Specifically, patients who had been living with diabetes for more than five years exhibited elevated odds of developing liver fibrosis. A study conducted by Luo et al. also demonstrated a positive correlation between diabetes duration and FIB-4 scores [20]. In line with this, a study conducted in Hong Kong revealed that liver-related events escalated linearly with the duration of diabetes [21]. This relationship can be attributed to extended exposure to diabetes, which leads to the accumulation of advanced glycation end products in the liver process that has been linked to the development of fibrosis [22].

Within our study, we found that individuals with diabetes who had higher levels of LDL cholesterol were at a heightened risk of developing liver fibrosis. These findings align with a prior study conducted by Kim et al., which also established a significant association between elevated LDL and liver fibrosis. Notably, the study by Kim et al. demonstrated that LDL levels increased alongside the severity of hepatic steatosis [23]. In a similar vein, our research also indicated that abnormal HDL cholesterol levels were linked to liver fibrosis. However, it's important to note that the relationship between HDL and LDL cholesterol and the risk of liver fibrosis is a topic of ongoing discussion within the medical community. Some studies suggest a lack of clear consensus on this matter and propose that HDL and LDL cholesterol alone may not be sufficient to predict liver fibrosis [24]. The intricacies of these lipid factors in liver fibrosis warrant further investigation and debate within the field.

Our study stands as a pioneering effort, being the first to use a questionnaire-based method to assess the risk of liver fibrosis among patients with diabetes. By doing so, it significantly contributes to the existing literature on the prevalence of liver fibrosis risk within this specific patient group. While there have been some studies aimed at evaluating liver fibrosis among patients with diabetes, their number remains relatively limited. Moreover, the unique feature of our current study is the utilization of a noninvasive technique, which holds the potential for replication in primary healthcare settings to efficiently identify individuals at a high risk of liver fibrosis among patients with diabetes.

Nevertheless, it's important to recognize certain limitations within our study. Notably, the screening method used in this research should not be viewed as a replacement for a clinician's diagnosis. As such, the findings should be interpreted with due caution. Additionally, our study adopted a cross-sectional design, which inherently restricts our ability to establish causal relationships between variables.

The prevalence of NAFLD is high among patients with diabetes. NAFLD comprises different stages, ranging from isolated steatosis to NASH. NASH is a chronic state of liver inflammation that leads to the transformation of hepatic stellate cells to myofibroblasts. These cells produce an extracellular matrix that results in liver fibrosis. So liver fibrosis can be considered as a chronic complication of NAFLD [25]. To streamline the implementation of NAFLD management, it has been incorporated into the flagship program of the Government of India for NCDs, specifically the *National Programme for Prevention and Control of Cancer, Diabetes, Cardiovascular Diseases, and Stroke* (NPCDCS), now known as NP-NCD. This program has provisions for fibroscan testing at district hospitals [10]. However, the utilization of the FIB-4 score at PHCs and Community Health Centers presents an opportunity to identify individuals at risk of NAFLD, enabling early intervention and referral. This approach can effectively alleviate the burden on district hospitals.

## Conclusions

The study provides valuable insights into the prevalence of liver fibrosis risk among Indian patients with diabetes, employing the FIB-4 risk score as a noninvasive tool. The prevalence of high risk of liver fibrosis among patients with diabetes was 4.6% (95% CI 2.7%-7.1%), whereas an intermediate risk of developing liver fibrosis was observed in 30.5%. The findings affirm the significance of age, diabetes duration, and lipid profiles, particularly LDL and HDL cholesterol, in predicting liver fibrosis risk within this population. The study advocates for integrating these screening tools into primary healthcare settings, alleviating the strain on larger healthcare facilities, and underscores the importance of early detection and management of liver fibrosis in patients with diabetes.

## References

[1] Anjana RM, Unnikrishnan R, Deepa M (2023). Metabolic non-communicable disease health report of India: the ICMR-INDIAB national cross-sectional study (ICMR-INDIAB-17). Lancet Diabetes Endocrinol.

[2] Younossi ZM, Koenig AB, Abdelatif D, Fazel Y, Henry L, Wymer M (2016). Global epidemiology of nonalcoholic fatty liver disease-meta-analytic assessment of prevalence, incidence, and outcomes. Hepatology.

[3] Duseja A, Singh SP, Saraswat VA (2015). Non-alcoholic fatty liver disease and metabolic syndrome-position paper of the Indian National Association for the study of the liver, Endocrine Society of India, Indian College of Cardiology and Indian Society of Gastroenterology. J Clin Exp Hepatol.

[4] Lee YH, Cho Y, Lee BW, Park CY, Lee DH, Cha BS, Rhee EJ (2019). Nonalcoholic fatty liver disease in diabetes. Part I: Epidemiology and diagnosis. Diabetes Metab J.

[5] De Silva NM, Borges MC, Hingorani AD (2019). Liver function and risk of type 2 diabetes: bidirectional Mendelian randomization study. Diabetes.

[6] Ballestri S, Zona S, Targher G (2016). Nonalcoholic fatty liver disease is associated with an almost twofold increased risk of incident type 2 diabetes and metabolic syndrome. Evidence from a systematic review and meta-analysis. J Gastroenterol Hepatol.

[7] Tarantino G, Finelli C (2013). What about non-alcoholic fatty liver disease as a new criterion to define metabolic syndrome?. World J Gastroenterol.

[8] Calzadilla Bertot L, Adams LA (2016). The natural course of non-alcoholic fatty liver disease. Int J Mol Sci.

[9] Tapper EB, Parikh ND (2023). Diagnosis and management of cirrhosis and its complications: a review. JAMA.

[10] (2023). Operational Guidelines of Non Alcoholic Fatty Liver Disease (NAFLD) Into NPCDCS. https://main.mohfw.gov.in/?q=newshighlights-42.

[11] Mózes FE, Lee JA, Selvaraj EA (2022). Diagnostic accuracy of non-invasive tests for advanced fibrosis in patients with NAFLD: an individual patient data meta-analysis. Gut.

[12] Sinha A, Bankura B (2023). Prevalence of nonalcoholic fatty liver disease in type 2 diabetes mellitus patients from the Eastern region of India. Diabetes Epidemiol Manag.

[13] Kuchay MS, Choudhary NS, Mishra SK (2021). Prevalence of clinically relevant liver fibrosis due to nonalcoholic fatty liver disease in Indian individuals with type 2 diabetes. JGH Open.

[14] Kawata N, Takahashi H, Iwane S (2021). FIB-4 index-based surveillance for advanced liver fibrosis in diabetes patients. Diabetol Int.

[15] Chung SM, Kang MK, Moon JS, Park JG (2023). Performance of simple fibrosis score in non-alcoholic fatty liver disease with and without type 2 diabetes. Endocrinol Metab (Seoul).

[16] Claudia M, Filozof CM, Jones S, Goldstein BJ (2018). Progression in liver fibrosis as assessed by the FIB-4 index in patients with type 2 diabetes. Diabetes.

[17] Lundholm MD, Bena J, Zhou K, Tsushima Y, Kashyap SR (2023). Prevalence and clinical determinants of non-alcoholic fatty liver disease by liver scores in adults with type 1 diabetes. J Diabetes Complications.

[18] Poynard T, Lebray P, Ingiliz P (2010). Prevalence of liver fibrosis and risk factors in a general population using non-invasive biomarkers (FibroTest). BMC Gastroenterol.

[19] Frith J, Jones D, Newton JL (2009). Chronic liver disease in an ageing population. Age Ageing.

[20] Luo Y, Wang C, Zhang T (2023). Factors associated with liver fibrosis in Chinese patients with type 2 diabetes mellitus and non-alcoholic fatty liver disease. Int J Gen Med.

[21] Zhang X, Wong GL, Yip TC (2022). Risk of liver-related events by age and diabetes duration in patients with diabetes and nonalcoholic fatty liver disease. Hepatology.

[22] Li X, Jiao Y, Xing Y, Gao P (2019). Diabetes mellitus and risk of hepatic fibrosis/cirrhosis. Biomed Res Int.

[23] Young Kim S, Mun S, Yu JH, Jin YJ, Ju Suh Y, Cho SH, Lee JW (2022). Association between small dense LDL levels and hepatic fibrosis in patients with nonalcoholic fatty liver disease. Medicine (Baltimore).

[24] Ganjooei NA, Jamialahmadi T, Nematy M (2021). The role of lipid profile as an independent predictor of non-alcoholic steatosis and steatohepatitis in morbidly obese patients. Front Cardiovasc Med.

[25] Heyens LJ, Busschots D, Koek GH, Robaeys G, Francque S (2021). Liver fibrosis in non-alcoholic fatty liver disease: from liver biopsy to non-invasive biomarkers in diagnosis and treatment. Front Med (Lausanne).

